# Nanoparticle-Based Targeted Drug Delivery Methods for Heart-Specific Distribution in Cardiovascular Therapy

**DOI:** 10.3390/pharmaceutics17111365

**Published:** 2025-10-22

**Authors:** Toshihiko Tashima

**Affiliations:** Tashima Laboratories of Arts and Sciences, 1239-5 Toriyama-cho, Kohoku-ku, Yokohama 222-0035, Kanagawa, Japan; tashima_lab@yahoo.co.jp

**Keywords:** cardiovascular diseases, heart-targeted drug delivery, nanoparticle-mediated delivery, receptor-mediated endocytosis/transcytosis

## Abstract

Cardiovascular diseases remain the leading cause of death worldwide and are often managed through invasive surgical procedures such as heart transplantation, ventricular assist device implantation, coronary artery bypass grafting, and stent placement. However, significant unmet medical needs persist in this field. The development of pharmaceutical agents using non-invasive delivery strategies is therefore of critical importance. Current treatments often target peripheral tissues or organs—such as capillary endothelial cells, vascular smooth muscle, and renal tubules—to reduce cardiac workload by lowering blood pressure. However, effective drug delivery directly to the myocardium continues to pose a significant challenge. For conditions such as congestive heart failure (CHF) and myocardial infarction (MI), targeted delivery of therapeutic agents to the heart is essential. In this perspective review, I discuss the potential and emerging strategies for non-invasive cardiac drug delivery, focusing on receptor-mediated endocytosis and transcytosis using nanoparticle-based delivery systems that have frequently been employed for targeting the brain or cancer cells although their use for cardiac delivery remains largely unexplored.

## 1. Introduction

**Figure 1:**
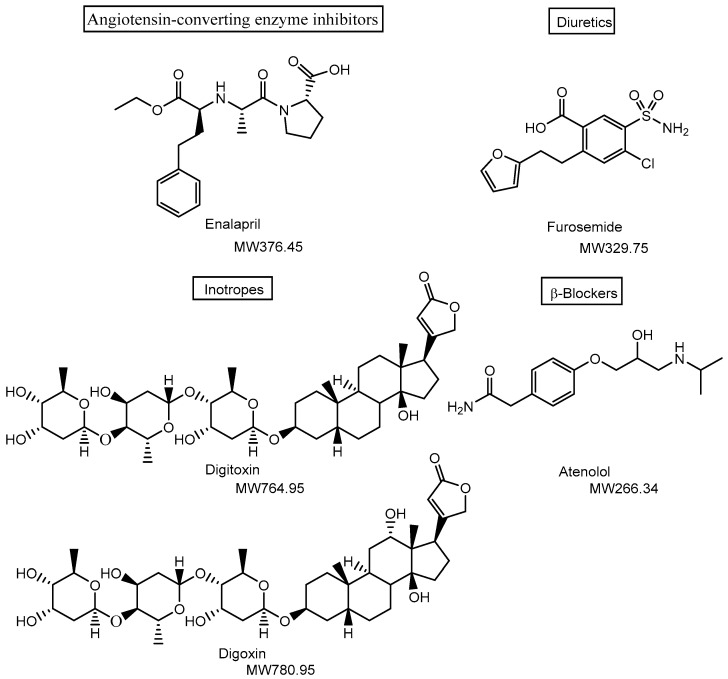
Structures of drugs used in the treatment of cardiovascular diseases. Medications such as angiotensin-converting enzyme inhibitors (e.g., enalapril) and diuretics (e.g., furosemide) are widely used to reduce cardiac workload by lowering blood pressure, primarily targeting tissues and organs such as capillary endothelial cells, vascular smooth muscle, and renal tubules. In contrast, digitoxin and digoxin act on the plasma membrane Na^+^/K^+^-ATPase of cardiac myocytes, directly targeting the heart. MW denotes molecular weight.

**Figure 2:**
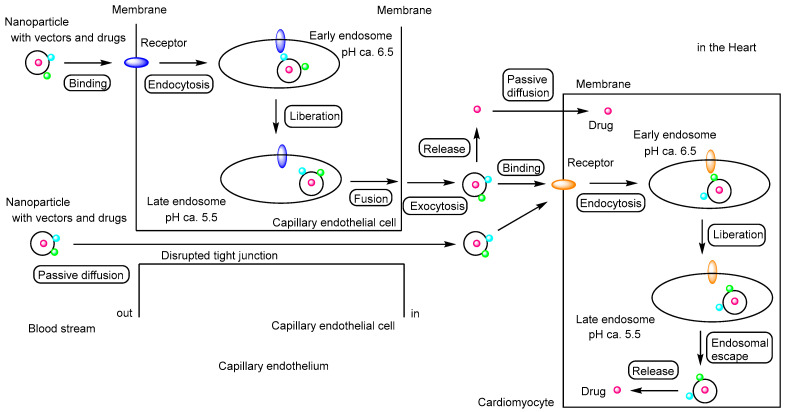
Schematic representation of the pathway by which drug-loaded nanoparticles, equipped with targeting vectors, exert their effects on cardiomyocytes, illustrating the concept of drug delivery specificity described in this review. Primarily, vector-functionalized nanoparticles cross the capillary endothelium via receptor-mediated transcytosis along the transcellular route. Subsequently, these nanoparticles enter cardiomyocytes through receptor-mediated endocytosis and release their cargo following endosomal escape. The released drugs then exert their biological activity. During myocardial infarction (MI) and chronic heart failure (CHF), disruption of tight junctions allows moderately sized nanoparticles to penetrate the capillary endothelium via the paracellular route. Additionally, drugs released into the cardiac interstitium may enter cardiomyocytes by passive diffusion. Red circles indicate drugs; light blue and green circles represent vectors; blue and orange ellipses indicate receptors.

Cardiovascular diseases—including coronary heart disease, cerebrovascular disease, peripheral arterial disease, rheumatic heart disease, congenital heart disease, deep vein thrombosis, pulmonary embolism, and congestive heart failure (CHF)—are the leading cause of death worldwide, accounting for an estimated 17.9 million deaths in 2019, of which 85% were due to heart attacks and strokes [1,2]. Therefore, in drug research and development, it is crucial to design pharmaceutical agents for the treatment of cardiovascular diseases using non-invasive approaches that do not rely on commonly performed surgical interventions such as heart transplantation, ventricular assist devices, bypass surgeries, or stent implantation. Despite advances in treatment, significant unmet medical needs remain in this therapeutic area. Clinically, medications such as angiotensin-converting enzyme inhibitors (e.g., enalapril), diuretics (e.g., furosemide), and others (Figure 1) are widely used to reduce cardiac workload by lowering blood pressure. These drugs target tissues or organs—such as capillary endothelial cells, vascular smooth muscle, and renal tubules—that are readily accessible through systemic circulation, even with oral administration. As such, they are well-suited for managing chronic conditions and preventing acute cardiovascular events. However, the primary target organ of inotropes such as digoxin (Figure 1), which increase heart rate, and β-blockers such as atenolol (Figure 1), which decrease heart rate, is the heart itself. These agents must be delivered to the heart via systemic circulation in most cases, although β-adrenergic receptors are also present in various non-cardiac tissues. Moreover, therapeutic drug monitoring is required to control blood concentrations of digitoxin, which can negatively impact patients’ quality of life. The shift toward nanoparticle-based delivery could be further driven by clinical needs and existing therapeutic challenges. Therefore, it is essential to develop drug delivery systems capable of selectively and efficiently transporting appropriate amounts of pharmaceutical agents to the heart, while minimizing off-target side effects and excessive pharmacological activity. In this perspective review, I discuss the potential and practical applications of non-invasive cardiac drug delivery via receptor-mediated endocytosis and carrier-mediated transport using transporters (Figure 2), based on nanodelivery systems grounded in structuralist principles [3,4].

## 2. Discussion

### 2.1. Strategies for Targeted Drug Delivery to the Heart

#### 2.1.1. Overview of Cardiac Anatomy

**Figure 3:**
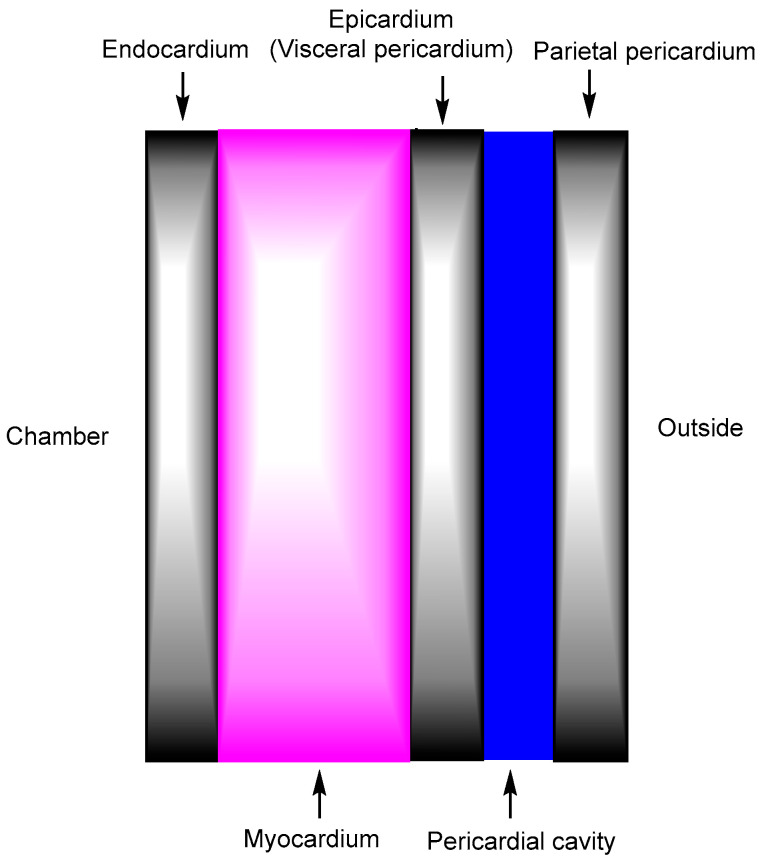
The heart wall is composed of three layers: the middle myocardium, the inner endocardium, and the outer epicardium. The pericardial cavity is the fluid-filled space located between the epicardium and the parietal pericardium. Drug-loaded nanoparticles administered intravenously should enter the heart through the myocardial capillary endothelium located in the mid-myocardium, rather than through the endocardial endothelium in the innermost layer of the endocardium, whose process likely occurs via receptor-mediated endocytosis or transcytosis through specific receptors expressed on the surface of mesh-patterned myocardial capillary endothelial cells.

The heart is a hollow, muscular organ that plays a vital role in the cardiovascular system. It is located behind the sternum and between the lungs. Structurally, the heart consists of four chambers: the right atrium, left atrium, right ventricle, and left ventricle [5]. The right atrium receives deoxygenated blood from the venae cavae and the coronary sinus. This blood is then passed to the right ventricle, which pumps it into the pulmonary trunk. The left atrium receives oxygenated blood from the pulmonary veins, which is subsequently transferred to the left ventricle. The left ventricle then pumps the arterial blood into the aorta for systemic circulation. The right atrium receives deoxygenated blood from the venae cavae and the coronary sinus. This blood is then transferred to the right ventricle, which pumps it into the pulmonary trunk. The left atrium receives oxygenated blood from the pulmonary veins, which is subsequently delivered to the left ventricle. The left ventricle then pumps this arterial blood into the aorta for systemic circulation. In general, vertebrate muscle tissue is classified into three types: skeletal muscle, smooth muscle, and cardiac muscle (myocardium). Among these, the myocardium, composed of cardiomyocytes, functions as a contractile syncytium responsible for generating the heart’s pumping force. It forms the middle layer of the heart wall, situated between the inner endocardium—a single-cell layer—and the outer epicardium (Figure 3) [6]. Coronary arteries, which originate from the aortic root and branch into smaller vessels, are formed by endocardial cells and supply nutrients and oxygen to the myocardium via the capillary endothelium. The endocardial endothelium and the myocardial capillary endothelium share common structural and functional features [7]. The myocardial capillary endothelium is continuous and characterized by tight junctions with an approximate diameter of 4 nm [8,9] Therefore, the transepithelial transport of most compounds occurs primarily via the transcellular route rather than the paracellular pathway. Transporters such as sodium/glucose co-transporter 1 (SGLT1) are expressed in cardiomyocytes and capillary endothelial cells, facilitating the uptake of hydrophilic, low-molecular-weight nutrients into the heart through their pore [10]. Nevertheless, transporter-focused studies on cardiac biology involving SGLT1 are not likely to be actively pursued. Endocytosis also takes place in the myocardial capillary endothelium to internalize macromolecules; for instance, insulin is likely internalized via receptor-mediated endocytosis involving the insulin receptor [11]. Consequently, intravenously administered substances intended for delivery to the heart must cross the capillary endothelium branching from the coronary arteries within the heart walls, rather than the inner endocardial layer.

#### 2.1.2. Non-Nanoparticle Drug Therapy Approaches

In general, orally administered cardiac drugs are transported from the systemic circulation into the heart across continuous capillary endothelial cells [12,13]. Therefore, digitoxin and digoxin, which are representative inotropes, must cross the capillary endothelium to reach cardiac tissue. Additionally, oral administration requires their absorption in the small intestine. While digitoxin undergoes hepatic metabolism, digoxin is primarily eliminated via renal excretion. Digoxin is known to be transported from systemic circulation into urine by the kidney through the human organic anion transporter OATP4C1 located on the basolateral membrane of proximal tubule cells (Km = 7.8 μM), followed by efflux mediated by multiple drug resistance 1 (MDR1, P-glycoprotein) at the apical membrane of these cells [14]. The uptake of digitoxin in the small intestine has been shown to be enhanced by inhibition of MDR1 [15]. In general, compounds are filtered from the glomerular capillaries into Bowman’s capsules, reabsorbed from Bowman’s capsules or renal tubules into the peritubular capillaries via active transport, secreted from the peritubular capillaries into the renal tubules, and ultimately excreted in the urine. The sequence of reabsorption and secretion may be reversed for certain compounds. Furthermore, intestinal absorption of digoxin in rats has been reported to be facilitated by organic anion transporting polypeptides (OATPs) and enhanced by GF120918, a specific MDR1 inhibitor [16]. These findings suggest that digitoxin and digoxin may cross cellular membranes not only via carrier-mediated transport involving OATP4C1 but also through passive diffusion, as MDR1 functions to actively efflux hydrophobic compounds that have passively diffused across the membrane [17].

Digitoxin and digoxin inhibit the Na^+^/K^+^-ATPase on the plasma membrane of cardiac myocytes, leading to intracellular Ca^2+^ accumulation through the secondary suppression of Na^+^/Ca^2+^ exchangers. As a result, calcium-induced calcium release via ryanodine receptors on the sarcoplasmic reticulum enhances myocardial contractile force [18]. The Na^+^/K^+^-ATPase is ubiquitously expressed in the plasma membranes of all animal cells [19]. In addition, although digitoxin and digoxin are substrates of MDR1, they are capable of crossing the capillary endothelium via passive diffusion. Consequently, these drugs may cause off-target side effects and severe toxicities due to overdose, necessitating routine monitoring of serum digoxin levels. To address this, the targeted delivery of cardiovascular drugs—including low-molecular-weight compounds—to the heart should be established using nanoparticle-based systems. Such systems, designed according to structuralist principles, can modulate material behavior within biological systems, including mechanisms such as receptor-mediated endocytosis and transcytosis.

#### 2.1.3. Nanoparticle Drug Therapy Approaches

Nanoparticle-based drug delivery systems have been developed to offer multiple benefits, including improved drug bioavailability and absorption, as well as the reduction of drug aggregation, enzymatic degradation, renal clearance, and undesired drug interactions [20]. A variety of materials with unique properties can be used to construct biocompatible, biodegradable, and controlled-release nanoparticles. These nanoparticles may be composed of: (i) synthetic biodegradable polymers, (ii) natural polymers such as chitosan, poly(lactic-co-glycolic acid) (PLGA), and poly(glycolic acid) (PGA), (iii) lipids including liposomes, micelles, and exosomes, (iv) inorganic materials such as gold (Au), silicon (Si), and magnetite (Fe_3_O_4_), (v) organic materials including albumin, monoclonal antibodies, and virosomes, (vi) emulsions, or (vii) other components (Table 1). Functionalized or engineered nanoparticles have been developed to exhibit various features, including: (i) passive targeting via the enhanced permeability and retention (EPR) effect, particularly in solid tumors [21,22]; (ii) active targeting through ligand–receptor interactions that induce receptor-mediated endocytosis; (iii) magnetic responsiveness; (iv) pH sensitivity; (v) thermosensitivity; (vi) enteric protection for oral administration; and (vii) other beneficial properties. Nanoparticles can be readily surface-modified with targeting vectors such as antibodies, tumor-homing peptides, and cell-penetrating peptides (CPPs). Currently, active targeting via receptor-mediated endocytosis or transcytosis, enabled by nanodelivery systems, is receiving considerable attention [23,24].

**Figure 4:**
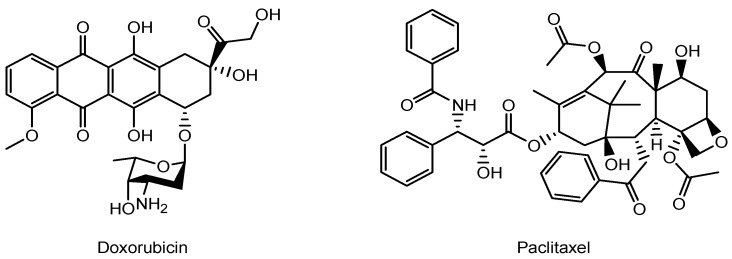
The structures of anti-cancer drugs.

Doxorubicin (Figure 4), an anthracycline antibiotic widely used in cancer treatment—along with paclitaxel—has been associated with the development of cardiomyopathy [25], MI, and CHF [26,27]. Although the precise mechanisms underlying doxorubicin-induced cardiomyopathy remain unclear, it is proposed that oxidative stress, enhanced by free radical generation, contributes to the progressive loss of myofibrils and vacuolization of myocardial cells. Notably, doxorubicin-loaded nanoparticles have been shown to reduce cell viability more effectively than free doxorubicin [28]. As a result, surgical intervention is required in some cases. Currently, no established drug therapy exists for doxorubicin-induced cardiomyopathy. To minimize doxorubicin distribution to the heart as a precautionary measure, doxorubicin-loaded nanoparticles based on lactosylated bovine serum albumin (BSA-Lac) were designed to target human liver cancer cells (HepG2) via receptor-mediated endocytosis through the asialoglycoprotein receptor (ASGPR), which is almost exclusively expressed and highly abundant in liver cells. In cytotoxicity assays, BSA-Lac-based nanoparticles carrying doxorubicin reduced cell viability more effectively than free doxorubicin, potentially reducing off-target cardiotoxicity in the treatment of hepatocellular carcinoma (HCC) [29]. ASGPR specifically interacts with galactose residues or lactose moieties [30]. Nonetheless, various pharmaceutical agents are under investigation as potential novel treatments for doxorubicin-induced cardiomyopathy, particularly those targeting left ventricular dysfunction or mitochondrial function. These agents include cannabidiol, carvedilol, hydrogen sulfide, coenzyme Q10, aldose reductase inhibitors, statins, angiotensin receptor blockers, α1-adrenergic receptor agonists, phosphodiesterase III (PDE-3) inhibitors, vitamin B1, monounsaturated fats (e.g., olive oil), phenolic antioxidants (e.g., oleuropein), and a range of bioactive compounds with hemodynamic, histological, and biochemical effects, such as flavonoids, hesperidin (a flavanone glycoside), pycnogenol, chrysin, acacetin, berberine, and cardamonin [31]. If antioxidant agents can be effectively and selectively delivered to the heart, non-invasive drug therapy may serve as an alternative to surgical intervention.

Nanoparticle-mediated drug delivery into the heart through the following examples (i)–(viii) is examined.

**Figure 5:**
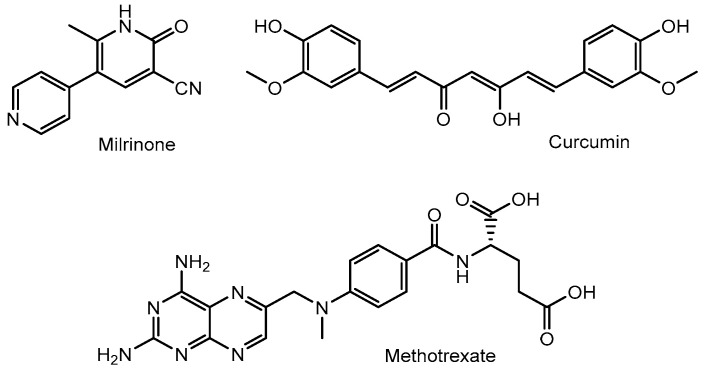
Structures of nanoparticle-delivered low-molecular-weight compounds such as milrinone, curcumin, and methotrexate.

**Figure 6:**
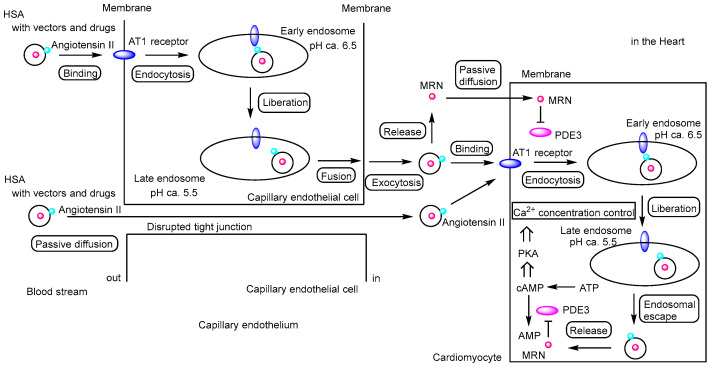
Schematic representation of the pathway by which milrinone (MRN)-loaded human serum albumin (HSA) nanoparticles exert their effects on cardiomyocytes. MRN-loaded HSA nanoparticles conjugated with angiotensin II (indicated by light blue circles) can enter the heart either via receptor-mediated transcellular transcytosis through angiotensin II type 1 (AT1) receptors (indicated by blue ellipses) or via paracellular passive diffusion through disrupted tight junctions between capillary endothelial cells. Once released in the heart, MRN may diffuse passively into cardiomyocytes. Alternatively, MRN may be released from endosomes into the cytosol after receptor-mediated endocytosis of angiotensin II–modified MRN-loaded HSA nanoparticles via AT_1_ receptors, although the mechanism of endosomal escape remains to be elucidated. MRN functions as a phosphodiesterase 3 (PDE3) inhibitor, preventing the hydrolysis of cyclic adenosine monophosphate (cAMP) into AMP. The accumulation of cAMP activates protein kinase A (PKA), which regulates cardiac contraction by modulating Ca^2+^ concentrations through sarcoplasmic reticulum Ca^2+^-ATPase (SERCA2a) and ryanodine receptor 2 (RyR2). Calcium uptake from the cytosol into the sarcoplasmic reticulum (SR) occurs via SERCA2a, whereas calcium release from the SR into the cytosol is mediated by RyR2. As a result, MRN induces mild enhancement of cardiac contraction. MRN is indicated by a red circle, and PDE3 is indicated by a magenta ellipse.

**Figure 7:**
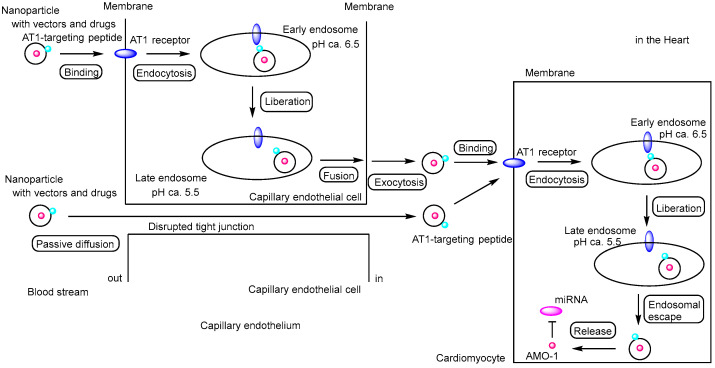
Schematic representation of the pathway by which anti-miR-1 oligonucleotide (AMO-1)–loaded nanoparticles exert their effects on cardiomyocytes. The nanoparticles protect the oligonucleotides from enzymatic degradation. The exact mechanism by which nanoparticles (AT1-PEG-DGL) cross the vascular endothelium remains unclear; however, receptor-mediated transcytosis via angiotensin II type 1 (AT1) receptors (indicated by blue ellipses) or paracellular passive diffusion through disrupted tight junctions between capillary endothelial cells are considered plausible pathways. Moreover, the mechanism of endosomal escape following AT_1_ receptor–mediated endocytosis also remains to be elucidated. AMO-1 is indicated by a red circle, and miRNA is represented by a magenta ellipse.

(i) Milrinone (MRN) (Figure 5) is a low-molecular-weight phosphodiesterase 3 (PDE3) inhibitor used in the treatment of CHF. Among the PDE3 isoforms, PDE3A—rather than PDE3B—regulates the inotropic response by hydrolyzing cyclic adenosine monophosphate (cAMP) and modulating protein kinase A (PKA) activity. Calcium uptake into the sarcoplasmic reticulum (SR) via SR Ca^2+^-ATPase (SERCA2a) is dependent on PKA-mediated phosphorylation of phospholamban (PLN) at Ser-16. However, excessive SR Ca^2+^ release, or “sparks”, can occur following the opening of ryanodine receptor 2 (RyR2), especially when hyperphosphorylated at sites such as Ser-2808 and Ser-2030 by PKA, or Ser-2814 by Ca^2+^/calmodulin-dependent protein kinase II (CaMKII). This dysregulation may contribute to cardiac arrhythmias or sudden cardiomyocyte death. Nevertheless, PDE3 inhibitors are also considered to have cytoprotective effects on the heart [32]. The angiotensin II type 1 (AT1) receptor, which specifically binds the angiotensin II peptide (an 8-amino acid sequence: DRVYIHPFHL), is overexpressed in the myocardium under conditions of MI and CHF [33,34]. MRN-loaded nanoparticles based on human serum albumin (HSA), surface-modified with angiotensin II through chemical conjugation using PA-(PEG)_4_-SPA, EDC, and sulfo-NHS, had an average diameter of 190.2 ± 5.7 nm. These nanoparticles were internalized into cardiac tissue via AT1 receptor-mediated endocytosis, resulting in a 3.92-fold higher concentration of MRN in the heart at 2 h post-injection, compared to free MRN. In a rat model of CHF, intravenous administration of these targeted nanoparticles significantly improved cardiac function, as evidenced by enhanced left ventricular fractional shortening (LVFS), relative to treatment with free MRN [35] (Figure 6). Clustering on the plasma membrane surface can induce endocytosis [36,37,38]. In the renin–angiotensin system, binding of angiotensin II to the AT1 receptor triggers intracellular signal transduction. In cardiac myocytes, AT1 receptor activation plays a role in processes such as cardiac fibroblast activation [39]. Clustering of AT1 receptors with nanoparticles may also promote receptor-mediated endocytosis. Additionally, it is known that HSA binds to the Gp60 receptor and to secreted protein acidic and rich in cysteine (SPARC). The Gp60 receptor is specifically expressed on the surface of continuous capillary endothelium in organs such as the heart and lungs, but not in the cortical brain [40]. SPARC, on the other hand, is expressed by various cell types including fibroblasts and endothelial cells, particularly in cancerous tissues [41]. Thus, HSA-based nanoparticles may be internalized into cardiac tissue via receptor-mediated, caveolae-dependent endocytosis through interactions with the Gp60 receptor or SPARC. However, no studies have yet demonstrated the delivery of HSA-based nanoparticles into the heart specifically via Gp60 receptor-mediated caveolae-dependent endocytosis. In contrast, angiotensin II has been shown to undergo AT1 receptor-mediated transcytosis across the BBB, resulting in its delivery to the brain [42]. This suggests that AT1 receptor-mediated transcytosis might also occur in the endothelial cells of the heart, although it remains unclear whether AT1 receptors are highly expressed in cardiac endothelial cells. Therefore, elucidating the expression status of AT1 receptors in cardiac endothelial cells is essential for establishing drug delivery through AT1 receptor-mediated transcytosis.

(ii) A specific microRNA-1 inhibitor—anti-miR-1 oligonucleotides (AMO-1)—suppresses cytosolic microRNA-1 expression in ischemic myocardium. MicroRNA-1 is a muscle-specific miRNA preferentially expressed in adult cardiac and skeletal muscle tissues [43], and it is implicated in various heart diseases. Intravenous administration of AMO-1 encapsulated in a nanovector (AT1-PEG-DGL) conjugated with an AT1-targeting peptide led to rapid accumulation in the infarcted myocardium, significantly more than in the control group lacking the AT1-targeting peptide. In an in vivo mouse model of MI, this targeted delivery system demonstrated a pronounced anti-apoptotic effect and reduced the myocardial infarct size by 64.1% compared to the MI control group [44]. Although the exact mechanism by which AT1-PEG-DGL crossed the vascular endothelium remains unclear (Figure 7), it was found that AT1 receptor expression peaked at 24 h post-MI. Angiotensin II is known to activate cardiac fibroblasts via the AT1 receptor [39,45], and AT1 receptor overexpression is thought to be a compensatory response to myocardial injury. However, the mechanism of endosomal escape following AT1 receptor-mediated endocytosis remains to be elucidated (Figure 7).

(iii) Oxidative stress and inflammation are elevated in CHF [46]. Curcumin (Figure 5), a compound with potent anti-inflammatory and antioxidant properties, has shown therapeutic potential in slowing the progression of CHF. Additionally, a graphene quantum dot-based electrochemical biosensor has been reported to exhibit high sensitivity, specificity, and selectivity for the early diagnosis of MI by detecting cardiac biomarkers such as troponin and myoglobin [47]. Indeed, PEGylated graphene quantum dot nanoparticles loaded with curcumin (Cur-PEG-GQDs) have been shown to reduce myocardial infarct size, fibrosis, and left ventricular end-diastolic pressure following intraperitoneal administration in an in vivo rat model of MI induced by ligation of the left anterior descending artery. Although curcumin decreased antioxidant markers and increased oxidant markers in the heart in a dose-dependent manner, non-classical mechanisms may contribute to the cardioprotective effects of Cur-PEG-GQDs against MI-induced cardiac dysfunction [48]. The mechanisms by which these nanoparticles are internalized into cardiac tissue from the systemic circulation remain unclear, despite the known association of graphene quantum dots with MI. Curcumin release from the nanoparticles is presumed to occur in either the extracellular or intracellular regions of myocardial cells or associated cell types. If curcumin is released in the extracellular space, it is likely that a portion enters cells primarily via passive diffusion [49]. Curcumin is not considered a substrate for specific transporters. It exhibits antioxidant and anti-inflammatory activities in myocardial cells [50], and also exerts anti-inflammatory effects in immune cells, including macrophages, which secrete pro-inflammatory cytokines such as IL-6, IL-1β, and TNF-α [51]. Therefore, extracellular release of curcumin may be advantageous for exerting its therapeutic effects. Furthermore, to overcome barriers to clinical translation by minimizing off-target effects in the brain and reducing hepatic clearance and sequestration, drug-loaded nanoparticles should ideally reach the myocardial capillary endothelium via the coronary circulation earlier than they reach the brain or liver. However, direct injection into the pulmonary veins or coronary arteries is clinically dangerous and impractical. Although intravenously administered nanoparticles must inevitably undergo first-pass metabolism and dilution in the lungs, all types of intravenous injections follow the circulation pathway—right atrium, right ventricle, pulmonary circulation, left atrium, left ventricle, and aorta—before reaching the coronary circulation, and thus arrive there earlier than in the brain or liver. The pulmonary first-pass effect may be attenuated through appropriate nanoparticle design.

(iv) Moreover, glycerol monooleate (GMO)-based biodegradable nanoparticles loaded with curcumin demonstrated greater protective effects compared to curcumin alone and more effectively enhanced the activity of antioxidant enzymes under both in vitro and in vivo conditions [52]. However, the mechanism by which GMO-based biodegradable nanoparticles are internalized remains unclear.

Therefore, curcumin-encapsulated nanoparticles are expected to serve as promising formulations for the treatment of cardiovascular diseases through multiple effective mechanisms.

(v) Methotrexate (MTX) (Figure 5) is an anti-inflammatory drug that inhibits not only dihydrofolate reductase but also AICAR (5-aminoimidazole-4-carboxamide ribonucleotide) transformylase. Inhibition of AICAR transformylase promotes the accumulation of AICAR, leading to increased adenosine release. This adenosine exerts anti-inflammatory effects through G-protein-coupled adenosine receptor subtypes, including A1, A2A, and A2B [53]. Moreover, activation of A2A and A2B receptors is essential for reducing infarct size in the hearts of wild-type C57Bl/6N mice [54]. While MTX uptake is mediated by folate receptors, apolipoprotein E (ApoE) is recognized by low-density lipoprotein (LDL) receptors on the cell membrane, triggering LDL receptor-mediated endocytosis. A lipid core nanoparticle (LDE) was formulated from a lipid mixture containing 100 mg of cholesteryl oleate and 200 mg of egg phosphatidylcholine. MTX-loaded LDE nanoparticles (LDE-MTX) significantly enhanced MTX uptake via the LDL receptor-mediated endocytic pathway in an in vitro assay using myocardial tissue from rats with MI, compared to MTX alone. In an in vivo study using MI-induced male Wistar rats, intravenous administration of LDE-MTX via the tail vein resulted in a 40% improvement in left ventricular (LV) systolic function, along with reduced cardiac dilation and LV mass [55]. Nonetheless, it has been demonstrated that LDE enters the heart from the systemic circulation via LDL receptor-mediated endocytosis. The release of MTX from the nanoparticles is presumed to occur either in the extracellular or intracellular regions of myocardial cells or associated cell types. If MTX is released extracellularly, a portion may enter cells through passive diffusion or via folate receptor-mediated transport.

(vi) MicroRNAs (miRNAs) play a significant role in the progression of MI. Bone marrow-derived mesenchymal stem cells (BMSCs), which are multipotent and possess homing ability, can migrate to sites of injury. Exosomes derived from BMSCs are commonly utilized as carriers for drug delivery. Murine hypoxia-conditioned BMSC-derived exosomes enriched with miR-125b-5p have been shown to promote ischemic cardiac repair by reducing cardiomyocyte apoptosis in MI mouse models, compared with murine normoxia-conditioned BMSC-derived exosomes enriched in miR-125b-5p [56]. Conversely, knockdown of miR-125b-5p in these exosomes exacerbates ischemic injury by increasing cardiomyocyte apoptosis. MiR-125b-5p inhibits apoptosis by downregulating the expression of p53 and BAK1. Furthermore, intravenous administration of ischemic myocardium-targeting peptide (IMTP)-conjugated hypoxia-conditioned BMSC-derived exosomes demonstrated higher specificity for ischemic lesions in the injured heart compared with scramble peptide-conjugated or intact hypoxia-conditioned BMSC-derived exosomes, as shown by Cy5.5 fluorescence imaging. Nonetheless, IMTP-conjugated hypoxia-conditioned BMSC-derived exosomes labeled with Cy5.5 were also detected in the liver and kidneys, in addition to the heart. Exosomes are believed to be internalized by recipient cells through membrane fusion and/or endocytosis [57]. Additionally, standardization challenges in exosome production include (a) source variability of parental cells, such as heterogeneity among BMSCs and differences in cell culture conditions; (b) inconsistencies in isolation and purification, including co-isolation of contaminants and scalability issues; (c) difficulties in characterization and quantification, such as inadequate reference standards and measurement variability; and (d) cargo heterogeneity and limited functional reproducibility, including variable molecular content, uncontrolled drug-loading efficiency, and batch-to-batch functional differences [58,59].

(vii) Gold–selenium core–shell nanostructures (AS-I/S NCs), modified with near-infrared II (NIR-II) photoacoustic imaging capability, an ischemic myocardium-targeting peptide (IMTP), and the mitochondrial-targeted antioxidant peptide SS31, significantly improved myocardial function and promoted angiogenesis in an in vivo rat model. NIR-II photoacoustic imaging indicated selective distribution to cardiomyocytes. IMTP facilitated targeted cellular uptake by cardiomyocytes, while SS31 enhanced antioxidant capacity by inhibiting reactive oxygen species (ROS), thereby reducing oxidative damage in H9c2 cells subjected to oxygen-glucose deprivation/reperfusion (OGD/R) injury [60]. No specific toxic effects attributable solely to selenium have been identified in many selenium nanoparticles, which differ from the AS-I/S NCs mentioned above [61]. Representative IMTPs, also referred to as cardiac-homing peptides, include peptide motifs such as CSTSMLKAC, CKPGTSSYC, and CPDRSVNNC, which have been identified from ischemic heart tissue. However, CSTSMLKAC conjugated to Sumo-mCherry accumulated not only in the ischemic myocardium but also in the kidneys [62]. Sumo-mCherry is a fusion protein composed of Small ubiquitin-like modifier (Sumo) and the red fluorescent protein mCherry. The molecular weight of the Sumo-mCherry-CSTSMLKAC conjugate is approximately 40 kDa. The cyclic nonapeptide CSTSMLKAC mimics endogenous peptide sequences found in proteins such as titin, optic atrophy 1 (OPA-1), and dynamin-related protein 1 (DRP-1). Although the cellular uptake mechanism of IMTPs remains unclear [63], the receptors for other tumor-homing peptides have been identified. For instance, RGD peptides bind to integrin αvβ3, while NGR peptides target the aminopeptidase N (APN/CD13) receptor. Nevertheless, IMTP-coated nanoparticles may facilitate targeted delivery to damaged cardiac tissue following heart injury.

(viii) 8P (PHWWEYRR), a peptide isolated from the stemless *Eleutherococcus senticosus*, is an antioxidant peptide that protects cells from oxidative stress-induced damage. PCM (WLSEAGPVVTVRALRGTGSW) is a myocardium-specific targeting peptide that binds selectively to tenascin-X on myocardial cells. In an in vivo rodent model of doxorubicin-induced MI, PCM-modified liposomes encapsulating 8P were effectively delivered to myocardial cells via tail-vein injection and exhibited antioxidant activity. Although this was an in vivo study conducted in rodents, prolonged survival and reduced arrhythmia incidence could be anticipated in humans as long-term functional outcomes of 8P-mediated oxidative stress prevention. Polyethylene glycol (PEG) modification extended the circulation time of the liposomes by evading immune recognition and clearance. Consequently, PEGylated liposomes may repeatedly circulate through the bloodstream and potentially cross the capillary endothelium into the heart tissue, although the exact mechanism of transendothelial transport remains unclear [64]. Nonetheless, the tight junctions of the cardiac capillary endothelium can be compromised under pathological conditions such as CHF and MI, due to factors including inflammation, ischemia, oxidative stress, and cytokine release [65,66,67,68]. This disruption of the endothelial barrier may facilitate the delivery of drugs and nanoparticles into the myocardial interstitial space, in a manner analogous to the EPR effect observed in solid tumors [21,22].

Accordingly, nanoparticle-based drug therapy can be achieved through receptor-mediated transcytosis using appropriate targeting vectors, such as the angiotensin II peptide, AT1-targeting peptide, or PCM (WLSEAGPVVTVRALRGTGSW).

### 2.2. Promising Strategies for Targeted Substance Delivery to the Heart

Tight junctions in the cardiac capillary endothelium are frequently disrupted in conditions such as CHF and MI, leading to increased vascular permeability. This enhanced permeability facilitates the passive accumulation of drug-loaded nanoparticles in cardiac tissue, providing a therapeutic window for targeted delivery. However, this process relies primarily on passive distribution. Incorporating active targeting strategies in addition to passive delivery may further enhance the efficiency of cardiac drug delivery. Nonetheless, the limited expression of specific surface proteins on cardiac capillary endothelial cells poses a challenge for effective active targeting.

Nanoparticle blood removal pathways (NBRPs) encompass various mechanisms responsible for the clearance and sequestration of nanoparticles from the bloodstream. These pathways are broadly categorized into cell-dependent mechanisms—(i) the reticuloendothelial system (RES), (ii) the mononuclear phagocyte system (MPS), (iii) other leukocyte-mediated processes, and (iv) additional cellular mechanisms—and cell-independent mechanisms, including (i) glomerular filtration, (ii) hepatic sinusoidal uptake, (iii) splenic sinusoidal filtration, and (iv) other non-cellular processes. As a result of these clearance mechanisms, nanoparticles tend to accumulate in the following order of organs: liver > spleen > lymph nodes > lungs > bone marrow > skin > kidneys > tumor [69]. Thus, effective nanoparticle delivery to the heart remains challenging. Notably, the AT1 receptor is among the most highly overexpressed receptors in the myocardium 24 h after MI and in CHF [44]. In damaged cardiomyocytes, certain substances may enter cells via passive diffusion through disruptions in the cell membrane. However, for more effective heart-targeted delivery via intravenous administration, selectively expressed receptors on the surface of cardiac capillary endothelial cells should be exploited. Nonetheless, no ideal heart-specific receptors have been identified to date. (i) Insulin receptors are ubiquitously expressed and are particularly abundant in the heart and endothelial cells [70]. It is well established that insulin receptors on brain capillary endothelial cells are exploited for drug delivery across the BBB via receptor-mediated transcytosis using anti-insulin receptor antibodies [71]. The fundamental machinery of receptor-mediated endocytosis is common to many tissues and organs, including the heart, brain, and cancer tissues. Receptor-mediated endocytosis and transcytosis for drug delivery across the BBB are well established. A similar strategy could enable drug-loaded nanoparticles to enter the heart via receptor-mediated endocytosis or transcytosis. By analogy, nanoparticles functionalized with anti-insulin receptor antibodies may similarly traverse the cardiac endothelium through receptor-mediated transcytosis, representing a potential strategy for targeted delivery to the heart.

(ii) Similarly, transferrin receptors on capillary endothelial cells at the BBB are utilized to mediate receptor-dependent endocytosis and transcytosis for delivering substances into the brain. However, it remains unclear whether transferrin receptors are highly expressed on capillary endothelial cells in the heart. In a study involving lipopolysaccharide (LPS) administration, transferrin receptor mRNA levels were significantly upregulated in the lungs but significantly downregulated in the hearts of rats 4 h post-treatment [72]. Surprisingly, transferrin conjugated with oligonucleotides has been successfully delivered to skeletal muscle and heart tissue via transferrin receptor 1 (TfR1)-mediated transport [73]. Although the expression levels of transferrin receptors on cardiac capillary endothelial cells remain unclear, cardiac myocytes are known to express transferrin receptors abundantly. Nevertheless, transferrin–oligonucleotide conjugates were able to cross both the capillary endothelium and the plasma membrane of cardiac myocytes. Given that iron is endogenously abundant in the heart, nanoparticles functionalized with anti-transferrin receptor antibodies may also traverse the cardiac endothelium via receptor-mediated transcytosis, offering a potential strategy for targeted delivery to cardiac tissue.

(iii) As a promising non-invasive accumulation strategy, magnetic nanoparticles composed of magnetic materials such as iron can be directed to target sites using an external magnetic field [74]. In cases where toxicity arises due to excessive accumulation at the target site, the external magnetic field can be easily discontinued, similar to external treatments such as compresses or medicated patches. Since iron is naturally present in the heart, iron-based nanoparticles are expected to exhibit minimal toxicity. Indeed, Fe_3_O_4_ core-based nanoparticles, functionalized with PEG linked via hydrazone bonds to antibodies targeting CD63 antigens on exosomes and to antibodies specific for myosin light chain surface markers on injured cardiomyocytes, accumulated at the site of a locally applied magnetic field. An external magnet attracts the magnetic vesicle shuttle to the infarcted area. Under the acidic pH of injured cardiac tissue in rabbit and rat models of MI, the hydrazone bonds were cleaved, resulting in the release of exosomes [75]. However, it was unclear how much more effectively the external magnet attracted the magnetic vesicle shuttle compared with the absence of a magnet. Moreover, monodisperse Fe_3_O_4_ nanospheres administered via tail vein injection were effectively localized in the myocardial tissue through external magnetic targeting in an in vivo experiment. The application of a magnetic field increased uptake by approximately 15-fold compared with conditions without a magnetic field [76]. Although most nanoparticles enter cells via endocytosis, the precise mechanisms by which these magnetic nanoparticles traverse the capillary endothelium of the heart remain unclear. It is well known that IgG antibodies and albumin exhibit prolonged half-lives due to their ability to evade lysosomal degradation through binding to the neonatal Fc receptor (FcRn) within endosomes. Upon endosomal acidification, free IgGs or albumin bind to FcRn, and subsequently dissociate and are released into the systemic circulation at physiological pH [77]. Thus, it is suggested that spontaneous endocytosis in capillary endothelial cells occurs more frequently than previously assumed, leading to the uptake of bystander substances such as antibodies and albumin. Consequently, magnetic nanoparticles accumulating near the heart may enter cardiac tissue as bystanders via spontaneous endocytosis or transcytosis. (iv) As an alternative strategy, graphene quantum dot nanoparticles carrying therapeutic cargos, as mentioned above, have been shown to improve outcomes in MI [47,78]. These nanoparticles can serve as carriers for highly selective drug delivery to the heart.

## 3. Conclusions

Cardiovascular diseases, also referred to as heart diseases, are serious disorders affecting the heart and blood vessels. In addition to pharmacological therapies aimed at regulating abnormal heart rhythms, lowering high blood pressure, or modifying myocardial contractility (e.g., inotropes), surgical interventions such as heart transplantation, ventricular assist device implantation, coronary artery bypass grafting, and stent placement are commonly employed. However, open-heart procedures impose significant physical stress on patients. Therefore, the development of innovative, less invasive treatment strategies for cardiovascular diseases is urgently needed.

Currently, tissue-selective drug delivery using nanoparticles has been explored, enabling the selective and preferential transport of therapeutic cargos to target sites by leveraging endogenous biological mechanisms. Although this approach may appear materialistic, it is grounded in structuralism [3,4], whereby materials function within the body under the regulation of complex biological systems. Biological activity is inherently coordinated. In the heart, nutrients are absorbed via carrier-mediated transport or receptor-mediated endocytosis. Oxygen and nutrients are supplied to cardiomyocytes from the coronary arteries through the myocardial capillary endothelium. Similarly, substance-loaded nanocarriers may also be delivered to the heart via these physiological pathways.

Nonetheless, in current research, the development of nanoparticles for cardiac drug delivery has primarily focused on their materials and composition. As a result, the number of successful demonstrations of nanoparticle-mediated drug delivery to the heart remains limited. The design of molecular vectors and the biological mechanisms underlying nanoparticle transport across the blood–brain barrier have been well studied to elucidate the time evolution of drug dosage and the saturation dynamics of cellular receptors, such as transferrin receptors. In contrast, the design of molecular vectors and the biological mechanisms governing nanoparticle transport to cardiac tissues and cells have not been thoroughly investigated. Consequently, both the time-dependent behavior of drug dosage and the receptor saturation rate remain largely unknown. Given the relatively small size of the heart, strategies for selective and efficient distribution—similar to those developed for brain-targeted delivery—are needed. Although nanoparticle-based drug delivery to the heart has been achieved, reports remain scarce due to the challenges associated with achieving such targeted and effective distribution. During conditions such as CHF and MI, disruption of tight junctions increases endothelial permeability, allowing nanoparticles or drug carriers to passively extravasate into the myocardial interstitial space. However, this represents a passive form of distribution, and the extent of tight junction disruption is highly dependent on the specific pathological conditions. Therefore, combining active targeting with passive distribution may enable more effective drug delivery to the heart. A widely studied strategy involves the use of well-designed nanoparticles functionalized with targeting ligands that bind to receptors on the surface of capillary endothelial cells, facilitating transendothelial transport via receptor-mediated transcytosis (Table 2). Following transcytosis, some nanoparticles are further internalized into cardiomyocytes through receptor-mediated endocytosis and subsequently release their cargos within endosomes or the cytosol. However, in many cases of cardiac delivery, the efficiency of endosomal escape remains poorly characterized. On the other hand, some nanoparticles release their cargos, which then enter cardiomyocytes or immune cells via passive diffusion or carrier-mediated transport.

Additionally, certain nanoparticles, such as graphene quantum dots, have been shown to cross the endothelium and accumulate in the infarcted heart, although the underlying mechanisms remain unclear. Magnetic nanoparticles composed of materials such as iron can be directed to target sites using an external magnetic field and subsequently enter the heart either via receptor-mediated transcytosis—when equipped with appropriate targeting vectors—or through passive diffusion facilitated by disrupted tight junctions. Ultimately, the development of well-designed nanoparticles guided by structuralist principles—where material behavior is regulated by biological machinery—holds great promise for achieving targeted drug delivery in the treatment of cardiovascular diseases.

## Figures and Tables

**Table 1:** Types of Nanoparticles Based on Material Composition.

#	Nanoparticle Formulations	Material Components
i	Synthetic biodegradable polymers	Poly(α-hydroxy esters), polyethylene glycol (PEG), polyurethane
ii	Natural polymers	Chitosan, poly(lactic-co-glycolic acid) (PLGA), poly(glycolic acid) (PGA)
iii	Vesicles (liposomes, micelles, exosomes)	Lipids
iv	Inorganic materials	Gold (Au), silicon (Si), magnetite (Fe_3_O_4_)
v	Organic materials	Albumin, monoclonal antibodies, virosomes
vi	Emulsions	Surfactants
vii	Other components	Others

**Table 2:** The introduced applications of drug delivery into the heart using nanoparticls as a carrier.

#	Formulation	Vector	Cargo	Status	References
i	MRN-loaded nanoparticles based on human serum albumin (HSA), surface-modified with angiotensin II	HSA, angiotensin II	MRN	Basic research	[35]
ii	AMO-1 encapsulated in a nanovector (AT1-PEG-DGL) conjugated with an AT1-targeting peptide	AT1-targeting peptide	AMO-1	Basic research	[44]
iii	PEGylated graphene quantum dot nanoparticles loaded with curcumin (Cur-PEG-GQDs)	Graphene quantum dot	Curcumin	Basic research	[48]
iv	Glycerol monooleate (GMO)-based biodegradable nanoparticles loaded with curcumin	GMO	Curcumin	Basic research	[52]
v	Methotrexate (MTX)-loaded lipid core nanoparticle (LDE) nanoparticles (LDE-MTX)	LDE against LDL receptor	MTX	Basic research	[55]
vi	Bone marrow-derived mesenchymal stem cell (BMSC)-derived exosomes enriched with miR-125b-5p	BMSC-derived exosomes	MiR-125b-5p	Basic research	[56]
vii	Gold–selenium core–shell nanostructures (AS-I/S NCs), modified with near-infrared II (NIR-II) photoacoustic imaging capability, ischemic myocardium-targeting peptide (IMTP), and the mitochondrial-targeted antioxidant peptide SS31	IMTP	Antioxidant peptide SS31	Basic research	[60]
viii	myocardium-specific targeting peptide PCM-modified liposomes encapsulating antioxidant peptide 8P	PCM	8P	Basic research	[64]
x	PEGylated graphene quantum dot nanoparticles loaded with MTX and MRN	Graphene quantum dot	MTX, MRN	Under analysis in Tashima lab	-

## Data Availability

No new data were created or analyzed in this study. Data sharing is not applicable to this article.
